# ICRF-assisted Box–Behnken design and optimization for rapid UPLC–PDA determination of dorzolamide hydrochloride and timolol maleate in an ophthalmic preparation

**DOI:** 10.1093/jaoacint/qsag060

**Published:** 2026-06-25

**Authors:** Erdal Dinç, Eda Büker

**Affiliations:** Ankara University, Department of Analytical Chemistry, Faculty of Pharmacy, Yenimahalle, Ankara 06560, Turkey; Gazi University, Department of Basic Pharmaceutical Sciences, Faculty of Pharmacy, Yenimahalle, Ankara 06330, Turkey

## Abstract

**Background:**

Simultaneous optimization of chromatographic resolution and analysis time constitutes a significant analytical challenge in multicomponent pharmaceutical analysis, as resolution-driven optimization strategies may improve peak separation without providing explicit control over retention time, often resulting in unnecessarily prolonged runtimes. To address this limitation, an optimization strategy based on a Box–Behnken experimental design was implemented in conjunction with the improved chromatographic response function (ICRF), which integrates separation quality and analysis time within a single mathematical objective function.

**Objective:**

This strategy describes the development of a rapid, chemometrically optimized Ultra Performance Liquid Chromatography coupled with a Photodiode Array Detector (UPLC–PDA) method for the simultaneous determination of dorzolamide hydrochloride (DH) and timolol maleate (TI) in a commercial ophthalmic preparation.

**Methods:**

A Box–Behnken experimental design and optimization approach was employed in combination with an ICRF, which integrates resolution, peak overlap, peak width, and runtime into a single composite objective function. This strategy enabled short runtime (or short retention time of analytes in a chromatogram) while preserving adequate peak separation.

**Results:**

Under the optimized conditions, complete chromatographic separation was achieved within 3 min using a BEH C18 column and a mobile phase consisting of acetonitrile–4 × 10^−4^ M CCl_3_COOH (60 + 40, v/v) at a flow rate of 0.32 mL/min with detection at 275 nm. The method demonstrated excellent linearity over the range of 5.0–40.0 µg/mL (*r* > 0.999), with LODs of 0.51 µg/mL for DH and 0.61 µg/mL for TI. Mean recoveries were 99.9% and 99.5% for DH and TI, respectively, with satisfactory precision and robustness.

**Conclusion:**

The proposed ICRF-assisted optimization approach provided high-resolution separation within minimal runtime and was successfully applied to the routine QC analysis of a commercial ophthalmic formulation.

**Highlights:**

The study demonstrates the effectiveness of composite response-based chemometric optimization in enhancing analytical efficiency in pharmaceutical drug analysis.

Glaucoma is a progressive optic neuropathy commonly associated with elevated intraocular pressure and remains one of the leading causes of irreversible blindness worldwide (1–3). Combination therapy using carbonic anhydrase inhibitors and β-blockers is widely employed to reduce aqueous humor production and control intraocular pressure. Dorzolamide hydrochloride (DH) and timolol maleate (TI) are frequently co-formulated in ophthalmic preparations for the management of open-angle glaucoma and ocular hypertension (4–7).

Several analytical methods have been reported for the determination of these compounds, including reverse-phase (RP)-HPLC, UPLC, spectrophotometry, and TLC–densitometry (8–17). However, the strong spectral overlap of DH and TI in the UV region limits the applicability of conventional spectrophotometric techniques for their simultaneous analysis. Moreover, official pharmacopeial procedures recommend separate analytical conditions for each active compound (18), resulting in increased analysis time and operational costs. Given the high sensitivity of ocular tissues and the low dosage levels of active ingredients, ophthalmic formulations require stringent QC. Therefore, rapid and reliable analytical methods are essential for routine QC of ophthalmic products.

Chromatographic method development is inherently a multivariable process in which resolution and analysis time must be considered simultaneously, as both are influenced by experimental parameters such as mobile phase composition, column temperature, and flow rate (19–23). In many experimental design-based optimization studies, resolution (R) is selected as the primary response variable. Although maximizing resolution ensures adequate separation, resolution-driven optimization does not explicitly control retention time and may lead to unnecessarily prolonged runtimes, thereby reducing overall analytical efficiency.

In multivariable chromatographic systems, where interaction (in the statistical sense of design of experiments [DoE]) effects among factors significantly influence peak separation, optimization based solely on a single response variable may not necessarily yield truly optimal analytical conditions. When retention time is not explicitly incorporated into the optimization criterion, the resulting stationary point may correspond to acceptable resolution but suboptimal runtime. Consequently, method validation performed under such conditions may not reflect the most efficient analytical performance. These limitations emphasize the need for composite response functions that integrate both separation quality and analysis time within a unified optimization framework.

Alternatively, desirability-based approaches are commonly used in chromatographic optimization, where multiple responses are transformed into dimensionless desirability values and combined via a geometric mean function (24,25). In such approaches, individual chromatographic responses remain mathematically independent and are only integrated after transformation. In contrast, the improved chromatographic response function (ICRF) integrates separation quality, analysis time, and peak broadening directly within a single mathematical expression, allowing these criteria to be evaluated simultaneously as an interdependent system. This unified structure enables direct and explicit control over chromatographic performance through adjustable coefficients, guiding the optimization process toward conditions that ensure both adequate resolution and minimal analysis time.

To address this limitation, composite chromatographic response functions have been introduced. Carle proposed a quantitative approach for evaluating peak overlap in chromatographic systems (26). Subsequently, ICRFs were developed to integrate key chromatographic parameters, including resolution, peak width, degree of overlap, and total analysis time, into a single objective function (27). By incorporating runtime directly into the optimization criterion, ICRF enables simultaneous control of separation efficiency and analysis duration. This approach has been applied in chemometric-assisted chromatographic method development, particularly in combination with response surface methodology and Box–Behnken experimental design (21–23, 28).

Accordingly, the present study aimed to develop and optimize a UPLC–PDA method for the simultaneous quantification of DH and TI in a commercial ophthalmic solution using a Box–Behnken design (BBD)-based chemometric optimization strategy. Optimal chromatographic conditions were established by minimizing the ICRF response, enabling high-resolution separation within a short analysis time. To the best of our knowledge, no previous study has reported the application of ICRF-based Box–Behnken optimization for the development of a UPLC–PDA method for the simultaneous determination of these compounds in ophthalmic preparations.

Previous studies have shown that overlapping chromatographic peaks can be resolved using advanced chemometric methods such as Parallel factor analysis (PARAFAC) and Three-way partial least squares (3W-PLS) without requiring full chromatographic separation (29). These approaches rely on post-acquisition multivariate data processing of co-eluted signals. In contrast, the present study focuses on chromatographic optimization to achieve physical separation within a short runtime. Therefore, the two approaches can be considered complementary rather than competing.

In this study, a BBD was employed to systematically investigate the effects of independent chromatographic variables, column temperature (X_1_), flow rate (X_2_), and mobile phase composition (X_3_), on chromatographic performance. The ICRF was selected as the dependent response variable, integrating resolution, peak width, degree of overlap, and total runtime into a single composite function. A second-order polynomial model was fitted to the experimental data obtained from the BBD matrix. The mathematical model describing the relationship between independent variables and the ICRF response can be expressed as follows:


(1) $$Y=\beta_{0}+\sum \beta_{i}X_{i}+\sum \beta_{\text{ii}}X_{i}{}^{2}+\sum \beta_{\text{ij}}X_{i}X_{j}$$

where Y = ICRF response; β terms = regression coefficients; and Xᵢ = coded independent variables. The mathematical resolution of the quadratic model was used to determine the optimal chromatographic conditions. This approach enabled the identification of the stationary point corresponding to the minimum ICRF value within the defined design space.

## Experimental

### Chemicals and reagents

HPLC-grade methanol and trichloroacetic acid (CCl_3_COOH) were purchased from Merck (Darmstadt, Germany). Reference standards of DH (99.3%) and TI (99.6%) were kindly supplied by World Medicine Pharmaceutical Industry (Istanbul, Turkey).

Commercial ophthalmic solution (Tomec^®^, labeled to contain 20 mg/mL DH and 5 mg/mL TI), manufactured by Abdi İbrahim Pharmaceutical Industry (Istanbul, Turkey), was purchased from a local pharmacy in Ankara, Turkey.

### Instrumentation and chromatographic conditions

Chromatographic analyses were performed using a Waters ACQUITY UPLC H-Class system (Waters, Milford, MA, USA) equipped with a quaternary solvent manager, cooled autosampler, column oven, and photodiode array (PDA) detector. Data acquisition and processing were carried out using Empower 2 software (Waters).

Separation was achieved on a Waters Acquity^®^ BEH C18 column (100 mm × 2.1 mm, 1.7 µm particle size). Ultrapure water was obtained from a Milli-Q purification system (Millipore, Bedford, MA, USA). Chromatographic conditions were optimized using a chemometric approach based on a Box–Behnken experimental design. The effects of column temperature, flow rate, and the concentration of CCl_3_COOH in the mobile phase were investigated as independent variables. Experimental design, response surface modeling, and optimization were performed using MATLAB (MathWorks Inc., Natick, MA, USA).

The optimized chromatographic conditions were as follows: a mobile phase of acetonitrile–4 × 10^−4^ M CCl_3_COOH solution (60 + 40, v/v), flow rate of 0.32 mL/min, column temperature of 45°C, injection volume of 1 µL, and detection at 275 nm. Under these conditions, complete separation of DH and TI was achieved within 3 min.

### Preparation of standard, validation, and design solutions

Stock solutions of DH and TI were prepared separately in methanol at a concentration of 100 µg/mL. Working calibration solutions were prepared over a concentration range of 5.0–40.0 µg/mL for both analytes.

For the Box–Behnken experimental design, mixed standard solutions containing 20 µg/mL of each analyte were prepared and analyzed under different chromatographic conditions according to the experimental matrix. These solutions were used to calculate the ICRF response values during the optimization process.

Validation solutions were prepared within the same concentration range. For accuracy studies, standard addition samples were prepared at three concentration levels (5.0, 20.0, and 32.0 µg/mL). For intra- and inter-day precision studies, three concentration levels (5.0, 20.0, and 35.0 µg/mL) were analyzed in triplicate. All solutions were freshly prepared on the day of analysis.

### Ophthalmic sample preparation

An aliquot of 1.2 mL commercial ophthalmic solution (Tomec) was transferred into a 100 mL volumetric flask and diluted to volume with methanol. The solution was stirred for 40 min and filtered through a 0.20 µm membrane filter (Sartorius Minisart, Hannover, Germany). Subsequently, 1.0 mL of the filtrate was further diluted to 10 mL with methanol. This procedure was repeated 10 times to assess reproducibility.

## Results and discussion

Chromatographic optimization of multicomponent systems requires simultaneous control of separation efficiency and analysis time. In the present study, the chromatographic behavior of DH and TI was systematically investigated using a multivariate experimental design approach. Rather than relying on conventional trial-and-error adjustments, a chemometric optimization strategy was employed to define the experimental domain and identify conditions that ensure both adequate resolution and minimal runtime. The optimized UPLC–PDA method was subsequently validated and applied to the analysis of a commercial ophthalmic formulation.

### Chemometric design and optimization

Prior to chemometric optimization, the effects of column temperature and flow rate on chromatographic separation and analyte elution behavior were evaluated, and these parameters were selected as significant factors for further optimization. Subsequently, different mobile phase systems containing various polarity-modifying additives were investigated. These experiments showed that variation in mobile phase polarity with different modifiers primarily affected retention times, without providing sufficient separation of the closely eluting peaks. In contrast, trichloroacetic acid was found to improve separation by enabling better resolution between closely eluting peaks. After concentration optimization, 4 × 10^−4^ M trichloroacetic acid was selected as the optimal mobile phase modifier.

Optimization of chromatographic systems involving multiple interacting variables requires a structured multivariate strategy rather than sequential parameter adjustment. In the present study, column temperature (X_1_), flow rate (X_2_), and the concentration of 4 × 10^−4^ M CCl_3_COOH in the mobile phase (X_3_) were identified as critical experimental factors influencing chromatographic performance. These variables were systematically investigated within a defined experimental domain using a three-level BBD (Table 1).

**Table 1:** Experimental ranges and coded levels of the independent variables in the BBD.

Level	4 × 10^−4^ M Cl_3_CCOOH, % (X_1_)	Column temperature, °C (X_2_)	Flow rate, mL/min (X_3_)
−1	12.0	30.0	0.20
0	38.0	45.0	0.30
1	64.0	60.0	0.40

The BBD generated 17 experimental runs, allowing systematic evaluation of linear, interaction, and quadratic effects of the selected chromatographic variables with a reduced number of experiments. Mixed standard solutions containing 20 µg/mL of each analyte were analyzed under the experimental conditions defined by the design matrix, and chromatograms were recorded at 275 nm (*see*  Figure 1).

**Figure 1:**
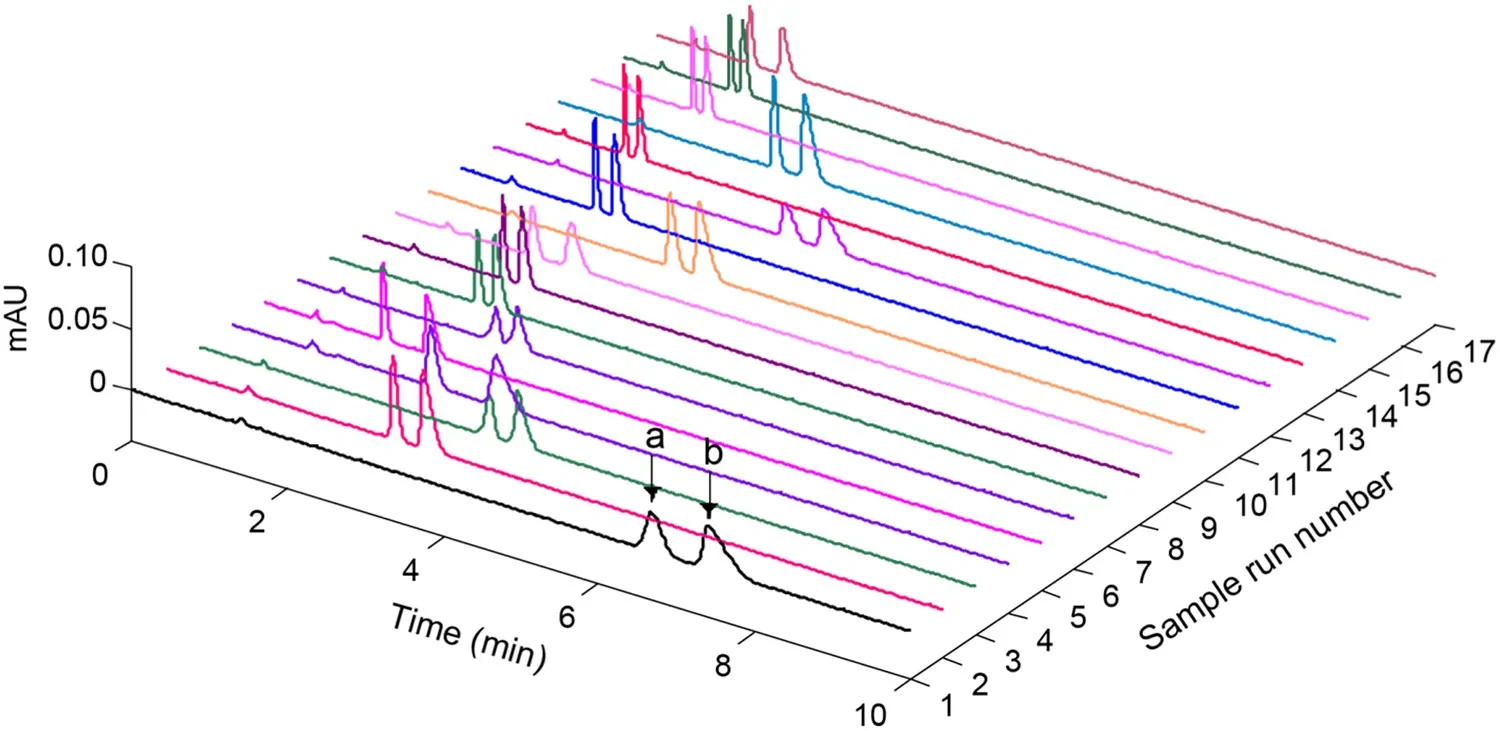
Representative UPLC chromatograms obtained under Box–Behnken experimental conditions at 275 nm for (a) DH and (b) TI.

The chromatographic data obtained from each experimental run were converted into response values using the ICRF, which served as the objective optimization criterion. The ICRF integrates separation efficiency and analysis time into a single composite function and is expressed as follows:


(2) $$\mathrm{ICRF}=(a(1-\frac{\sum_{i=1}^{N-1}\theta_{s,l}}{N-1})+1)(1+{\left(\frac{t_{f}}{t_{\mathrm{opt}}}\right)}^{b}){\left(1+\frac{\sum w_{i}}{N}\right)}^{c}$$

where N = expected number of peaks; *t_f_* = retention time of the last eluting peak; *t_opt_* = selected optimal separation time; *w_i_* = baseline peak width; and *a*, *b*, and *c* = weighting coefficients controlling the relative contributions of overlap, runtime, and peak broadening terms, respectively.

In the present study, the coefficients *a*, *b*, and *c* were systematically adjusted during the optimization process, and the optimal values were determined as *a* = 1, *b* = 2, and *c* = 1. This selection ensured that chromatographic conditions provided complete separation (resolution >2) while minimizing total analysis time. The higher weighting assigned to the runtime term (b) reflects the deliberate emphasis on reducing analysis time while maintaining adequate chromatographic resolution.

The term θ_*s*_, _l_ describes the degree of peak overlap and was calculated according to Carle’s approach (26):


(3) $$\theta =1-\frac{H_{v}|t_{R,l}-t_{R,s}|}{\left|t_{R,v}-t_{R,s}|(H_{l}-H_{s})+H_{s}(t_{R,l}-t_{R,s}\right)}$$

where *H_s_* and *H_l_* = peak heights of the smaller and larger peaks; *H_v_* = valley height at the point of intersection; *t_R,,s_* and *t_R,l_* = corresponding retention times; and *t_R,v_* = valley time. Minimization of the ICRF response ensured simultaneous reduction of peak overlap and runtime while maintaining adequate chromatographic efficiency within the experimental design space.

ICRF was employed as a composite objective function to integrate separation efficiency and analytical time into a single response. The function incorporates parameters related to peak resolution, baseline peak width, degree of overlap, and retention time of the last eluting peak. Unlike conventional resolution-based optimization, the mathematical structure of ICRF inherently penalizes excessive runtime while maintaining adequate separation. Peak overlap was quantified according to Carle’s approach (26). Consequently, minimization of the ICRF value enabled identification of chromatographic conditions providing high-resolution separation within the shortest possible analysis time.

Unlike desirability-based approaches that combine independent responses after transformation, the ICRF directly penalizes chromatographic conditions associated with excessive runtime or peak broadening within a single objective function. This approach ensures that the optimization process is inherently directed toward achieving both high resolution and minimal analysis time simultaneously.

### Response surface analysis

Three-dimensional response surface plots were generated to visualize the interaction effects of the selected chromatographic variables on the ICRF response (Figure 2). These plots provide a graphical interpretation of the quadratic model obtained from the BBD and illustrate the combined influence of mobile phase composition (X_1_), column temperature (X_2_), and flow rate (X_3_) on overall chromatographic performance.

**Figure 2:**
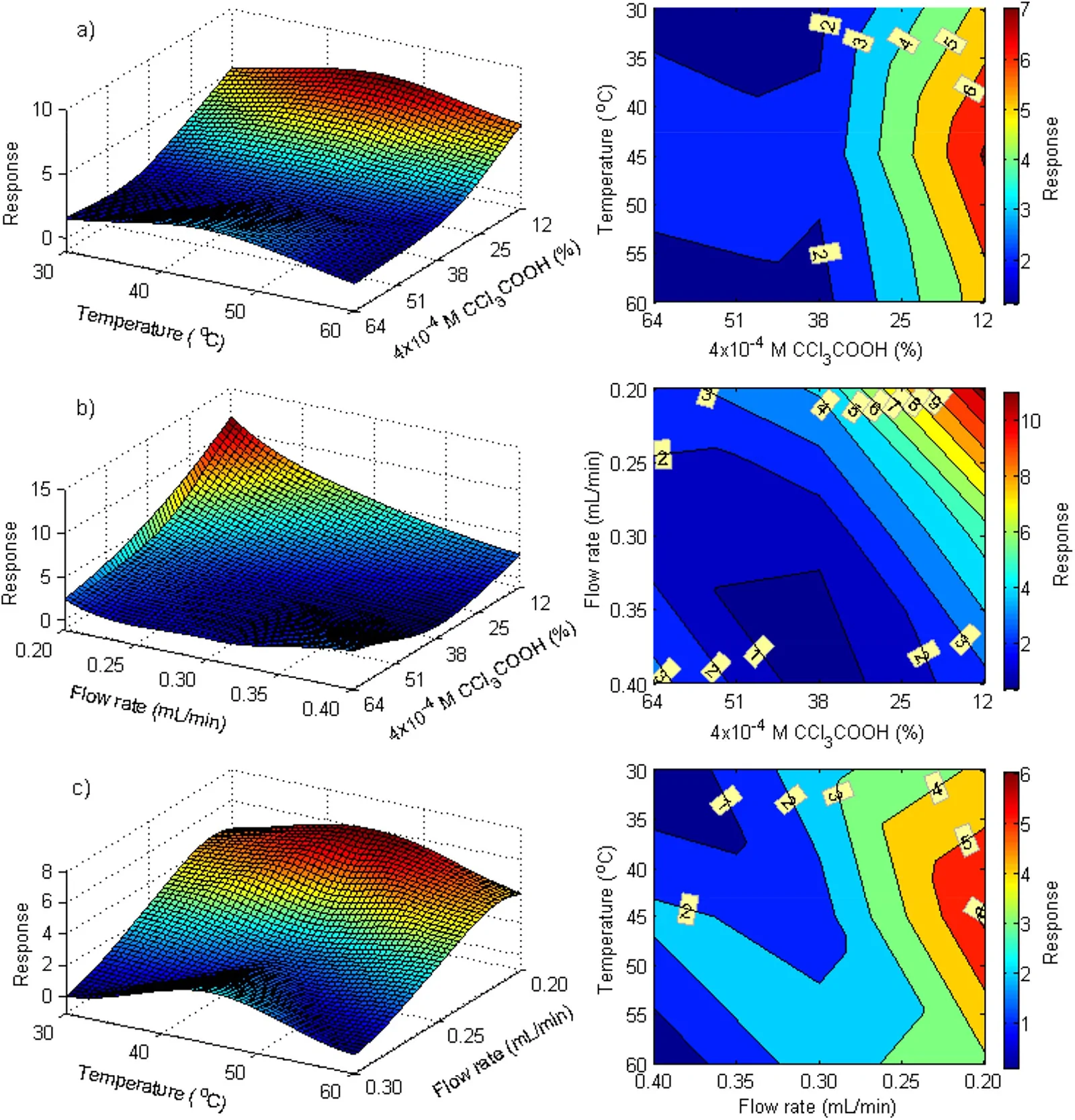
Response surface analysis of the ICRF. (a) Effect of column temperature and CCl_3_COOH concentration; (b) effect of flow rate and CCl_3_COOH concentration; and (c) three-dimensional surface plot and corresponding contour map showing the combined (interaction) effect of flow rate and acid concentration on the ICRF value.

The relationship between the independent chromatographic variables and the ICRF response was described using a quadratic response surface model obtained through multiple regression analysis. This second-order polynomial model captures the combined linear, quadratic, and interaction effects of the coded experimental factors on the composite chromatographic response. The fitted model is expressed as follows:


(4) $$y=1.554-2.0691x_{1}-0.0655x_{2}-1.9604x_{3}+2.4041x_{1}^{2}-0.5529x_{2}^{2}+1.0869x_{3}^{2}-0.1685x_{1}x_{2}+2.4343x_{1}x_{3}+0.1165x_{2}x_{3}$$

where *y* = predicted ICRF value and X_1_, X_2_, and X_3_ =coded levels of the investigated factors. The linear terms describe the primary influence of each factor, the quadratic terms account for curvature effects within the design space, and the cross-product terms reflect pairwise interactions between variables. The magnitude and sign of the regression coefficients provide insight into the direction and relative strength of each factor’s contribution to the overall chromatographic response.

The quadratic regression model was constructed by fitting the coded independent variables (X_1_, X_2_, and X_3_) to the ICRF response. The optimal values of factors (X_1_, X_2_, and X_3_) were subsequently determined from the mathematical solution of Equation (3), yielding the stationary point corresponding to the minimum ICRF value. Statistical significance tests for the regression coefficients and model adequacy tests for the quadratic regression model were conducted and summarized in Table 2.

The response surfaces clearly indicate that ICRF values are highly sensitive to the interaction between flow rate and acid concentration, as well as between flow rate and column temperature. Regions corresponding to elevated ICRF values were associated with reduced flow rates and lower proportions of CCl_3_COOH, reflecting increased analysis time and/or compromised separation efficiency. Conversely, lower ICRF values were observed within a defined intermediate domain of temperature and flow rate, indicating balanced chromatographic conditions.

The contour maps further confirm the presence of significant interaction effects, demonstrating that optimal chromatographic performance cannot be achieved by adjusting a single factor independently. Instead, simultaneous optimization of the variables within the design space is required.

Minimization of the ICRF response led to the identification of the optimal chromatographic conditions: column temperature of 45°C, flow rate of 0.32 mL/min, and a mobile phase of acetonitrile–4 × 10^−4^ M CCl_3_COOH (60 + 40, v/v). Under these conditions, high-resolution separation was achieved within a short analysis time, confirming the effectiveness of the response-based chemometric optimization strategy.

A supplementary desirability-based evaluation was also performed using chromatographic resolution (R) and final elution time (*t_f_*) as response variables. In this approach, resolution was maximized while final elution time was minimized. The overall desirability function was calculated according to


$$D={\left(d_{R}\;x\;d_{t}\right)}^{1/2}$$

where *d_R_* and *d_t_* represent the individual desirability values for resolution and final elution time, respectively. The final elution time *(t_f_*) was defined as the retention time of the last eluting peak, since chromatographic separation is practically completed after elution of the final analyte peak.

Based on the experimental data, the highest desirability was obtained for Run 11 (Table 3), corresponding to a 64% mobile phase composition that was mixture of acetonitrile and 4 × 10⁻⁴ M CCl₃COOH (60:40, v/v), a 60°C column temperature, and a 0.30 mL/min flow rate, with R = 1.7778 and *t_f_ *= 2.4192 min. Both the desirability- and ICRF-based evaluations indicated similar optimal chromatographic regions, characterized by higher column temperatures and optimized mobile phase compositions. Under the optimized ICRF conditions, the chromatographic peaks of DH and TI were observed at approximately 1.62 and 1.86 min, respectively, demonstrating that effective chromatographic separation was achieved within 2 min. However, the nominal total run time was maintained below 3 min to allow adequate column re-equilibration and ensure stable chromatographic performance between injections.

**Table 2:** Statistical significance of regression coefficients and model adequacy tests for the quadratic regression model.

Parameters	Coefficient	*t*-Test	*P*-value
Intercept	1.5547	28.30	0.0000
X_1_	−2.0691	−79.41	0.0000
X_2_	−0.0655	−2.81	0.0262
X_3_	−1.9604	−84.12	0.0000
x_1_^2^	2.4041	63.50	0.0000
X_2_^2^	−0.5529	−13.00	0.0000
X_3_^2^	1.0869	25.54	0.0000
X_1_*X_2_	−0.1685	−4.57	0.0026
X_1_*X_3_	2.4343	66.06	0.0000
X_2_*X_3_	0.1165	3.16	0.0159

**Table 3:** Box–Behnken experimental design matrix and corresponding observed and predicted ICRF values.

Experiment number	4 × 10^−4^ M Cl_3_CCOOH	Column temperature	Flow rate	**ICRF** ^a^	
	X_1_ (%) (coded)	X_2_ (^0^C) (coded)	X_3_ (mL/min) (coded)	Observed	Predicted
1	−1	0	−1	11.6165	11.5095
2	0	0	0	1.5560	1.5547
3	1	−1	0	1.5874	1.5707
4	0	−1	−1	4.1937	4.2310
5	−1	−1	0	5.3232	5.3719
6	1	0	−1	2.5443	2.5028
7	0	0	−1	4.5346	4.6020
8	0	1	0	0.9576	0.9362
9	−1	1	0	5.5375	5.5779
10	0	1	1	0.2173	0.1793
11	1	1	0	1.1277	1.1028
12	−1	0	1	2.7023	2.7201
13	0	1	−1	3.8233	3.8671
14	0	0	1	0.6999	0.6812
15	0	−1	1	0.1216	0.0772
16	0	−1	0	1.0920	1.0672
17	1	0	1	3.3672	3.4505

a ICRF = Improved chromatographic response function.

### Method validation and quantitative performance

The linearity of the optimized UPLC–PDA method was evaluated using 25 calibration solutions within a concentration range of 5.0–40.0 µg/mL for both analytes. Calibration curves were constructed by plotting peak area versus concentration at 275 nm. Retention times were 1.62 min for DH and 1.86 min for TI under optimized chromatographic conditions (Figure 3). Linear regression analysis was performed to establish the calibration equations, and statistical parameters, including slope, intercept, and correlation coefficients, are summarized in Table 4.

**Figure 3:**
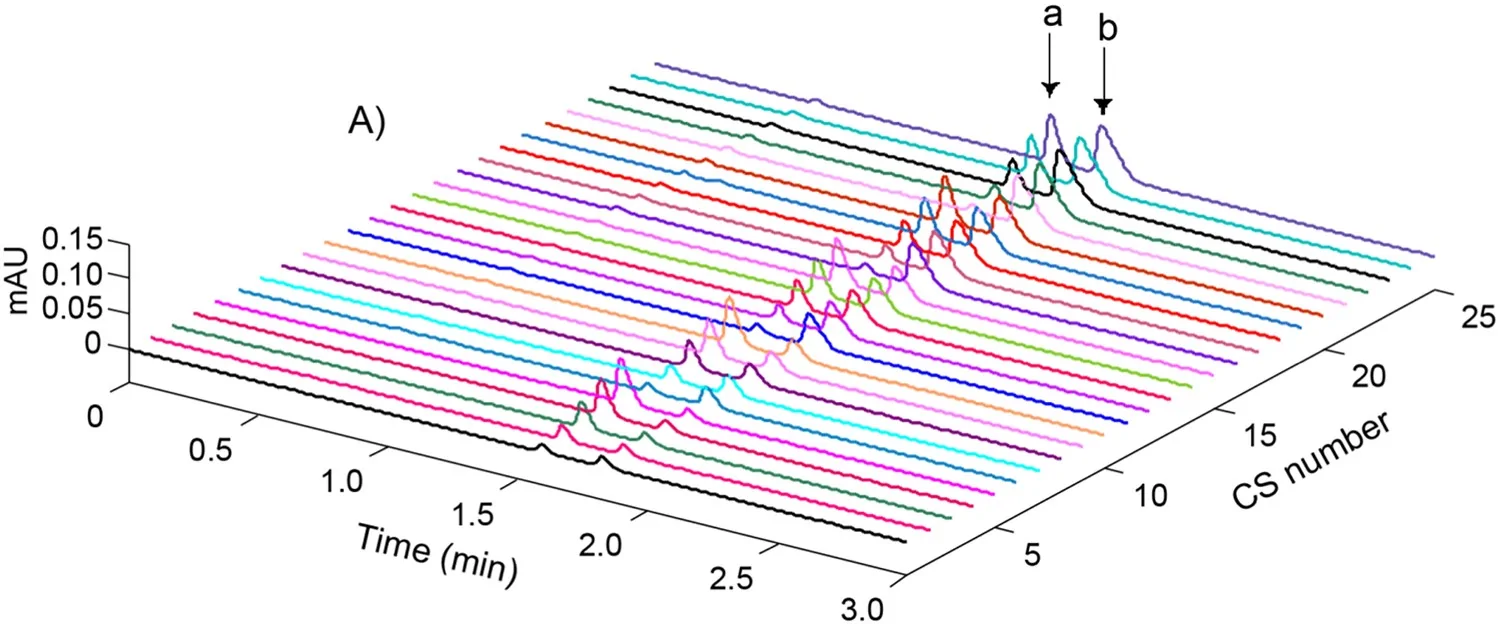
UPLC chromatograms of calibration samples recorded at 275 nm. Retention times of (a) DH and (b) TI are indicated.

**Table 4:** Linear regression and validation parameters for DH and TI.

Parameter	DH	TI
m^a^	1.0619	1.7773
n^b^	0.1681	0.9884
*r* ^c^	0.9994	0.9992
SD (m)^d^	7.35 × 10^−3^	1.46 × 10^−2^
SD (n)^e^	1.81 × 10^−1^	3.60 × 10^−1^
SD (*r*)^f^	1.44 × 10^−3^	1.72 × 10^−3^
LOD, μg/mL	0.51	0.61
LOQ, μg/mL	1.70	2.02

a m = Slope.

b n = Intercept.

c *r* = Correlation coefficient.

d SD (m) = Standard deviation of slope.

e SD (n) =Standard deviation of intercept.

f SD (*r*) = Standard deviation of correlation coefficient.

Method validation was conducted to assess accuracy, precision, and reliability. A validation set comprising 10 mixed standard solutions within the working range was analyzed. Mean recoveries were 99.9% for DH and 99.5% for TI, with RSD values below 2.5% (Table 5), confirming satisfactory accuracy and repeatability.

**Table 5:** Recovery results for DH and TI obtained using the optimized UPLC–PDA method.

		Added (µg/mL)		Found (µg/mL)		Recovery (%)	
Mixture number	DH		TI	DH	TI	DH	TI
1	32.0		5.0	32.80	4.96	102.5	99.1
2	32.0		10.0	32.95	10.20	103.0	102.0
3	32.0		20.0	32.93	20.65	102.9	103.3
4	32.0		30.0	32.40	30.53	101.3	101.8
5	32.0		40.0	32.40	40.35	101.2	100.9
6	5.0		8.0	4.94	8.01	98.7	100.1
7	10.0		8.0	10.16	7.89	101.6	98.6
8	20.0		8.0	20.73	8.06	103.6	100.7
9	30.0		8.0	30.30	7.85	101.0	98.1
10	40.0		8.0	40.56	7.77	101.4	97.1
					Mean	99.9	99.5
					SD	2.50	2.14
					RSD	2.50	2.15

Intra- and inter-day precision were evaluated at three concentration levels (5.0, 20.0, and 35.0 µg/mL). Analyses were performed in triplicate on the same day and over six consecutive days. The obtained recovery, RSD, and relative standard error values (Table 6) demonstrated consistent method performance and intermediate precision.

**Table 6:** Intra-day and inter-day precision and recovery results for DH and TI.^a^

Precision	Added (µg/mL)		Found (µg/mL)		Recovery (%)		RSD (%)		RSE (%)^b^	
	DH	TI	DH	TI	DH	TI	DH	TI	DH	TI
Intra-day	5.0	5.0	5.10	5.05	101.9	101.1	2.33	1.62	1.95	1.08
	20.0	20.0	20.49	20.73	102.5	103.7	0.71	4.93	2.47	3.65
	35.0	35.0	35.15	36.03	100.4	102.9	1.37	2.19	0.44	2.94
Inter-day	5.0	5.0	5.12	5.12	102.4	102.4	3.91	2.36	2.36	2.42
	20.0	20.0	20.45	20.03	102.3	100.1	1.45	1.50	2.27	0.13
	35.0	35.0	34.89	34.81	99.7	99.4	0.60	0.63	−0.33	−0.56

a n = 3.

b RSE = Relative standard error.

The applicability of the method to commercial ophthalmic formulations was examined using the standard addition technique to evaluate potential excipient interference. Sample preparation and analysis were performed under optimized chemometric conditions. The added amounts were calculated by subtracting the formulation contribution from the change in total measured concentrations. Recovery values and associated RSD results (Table 7) confirmed the absence of significant matrix effects and demonstrated the suitability of the method for routine QC analysis. While a conventional International Council for Harmonisation (ICH)-based robustness study was not performed as a separate validation step, the DoE-based optimization approach employed in this study enables simultaneous assessment of multiple method parameters. It provides insight into method robustness within the experimental design space. However, the robustness evaluation reported herein reflects the stability of the optimized chromatographic region within the experimental design space and should not be interpreted as a full ICH-style robustness validation study.

**Table 7:** Results of standard addition analysis for DH and TI using the optimized UPLC–PDA method.^a^

Commercial ophthalmic formulation	Added (µg/mL)		Found (µg/mL)		Recovery (%)		RSD^b^ (%)	
	DH	TI	DH	TI	DH	TI	DH	TI
Formulation	5	5	5.12	5.05	102.4	101.0	0.12	0.05
Formulation	20	20	19.86	20.68	99.3	103.4	0.78	0.23
Formulation	32	32	32.19	32.93	100.6	102.9	0.60	0.52

a n = 3.

b RSD = Relative standard deviation.

### System suitability assessment

System suitability parameters were evaluated to confirm the adequacy of the optimized UPLC–PDA method. Selectivity, resolution, peak asymmetry, tailing factor, and theoretical plate number were calculated from chromatograms obtained under optimized conditions (Table 8).

**Table 8:** System suitability parameters for the optimized UPLC–PDA method.

Parameters	Found	Reference
Sensitivity	2.12	>2
Resolution	3.17	>2
Peak asymmetry	1.26	0.9–1.4
Tailing factor	1.66	<2
Theoretical number of layers	7755	>2000

All calculated parameters met the recommended acceptance criteria, demonstrating satisfactory chromatographic performance and confirming the reliability of the method for quantitative analysis of DH and TI.

### Application to commercial formulation

The optimized UPLC–PDA method was applied to the analysis of a commercial ophthalmic formulation (Tomec). Sample preparation was performed as described in the *Experimental* section. Chromatograms were recorded under optimized conditions, and analyte concentrations were calculated using the established calibration equations.

Representative chromatograms are shown in Figure 4, demonstrating clear separation of DH and TI in the formulation matrix. The assay results are summarized in Table 9 and were found to be consistent with the labeled amounts, confirming the applicability of the method for routine QC analysis.

**Figure 4:**
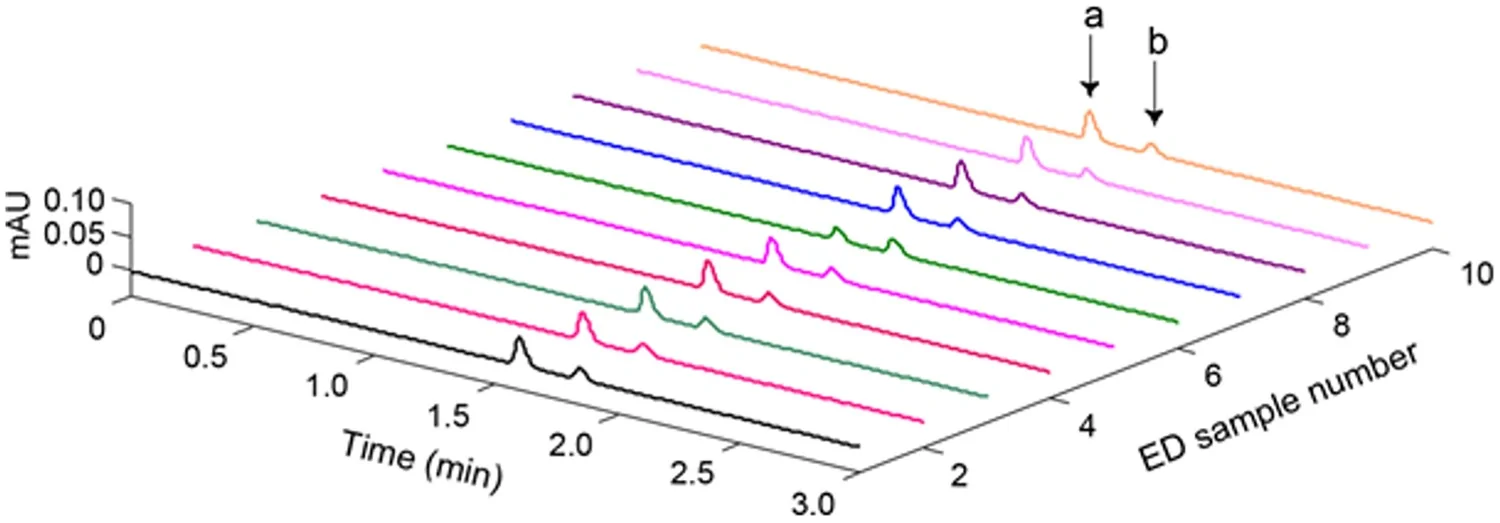
UPLC chromatograms of a commercial ophthalmic formulation obtained under optimized chromatographic conditions at 275 nm for (a) DH and (b) TI.

**Table 9:** Assay results for DH and TI in a commercial ophthalmic formulation using the optimized UPLC–PDA method. (Label claim: DH 20.0 mg/mL; TI 5.0 mg/mL.)

Experiment number	Concentration (mg/mL)	
	DH	TI
1	19.84	5.20
2	20.43	5.24
3	20.50	5.09
4	20.26	5.00
5	19.66	4.96
6	19.48	5.22
7	19.64	4.93
8	20.60	5.31
9	20.28	5.06
10	20.62	5.12
Mean	20.13	5.11
SD	0.43	0.13
RSD	2.15	2.49

A comparison with previously reported chromatographic methods is presented in Table 10. Compared to these, including UPLC-based approaches such as that of Sharma et al., which require a total run time of 30 min for impurity profiling, the proposed method significantly reduces the analysis time while maintaining efficient chromatographic separation. This highlights the advantage of the optimized ICRF-based approach in achieving rapid and effective analysis.

**Table 10:** Comparison of reported analytical methods with the proposed optimized UPLC method.

Method	Column	Mobile phase	λ (nm)	Retention time DH/TI (min)	Analysis time (min)	Remarks	Ref
UPLC	C18	Phosphate buffer (pH 2.6)/MeOH–ACN–H₂O (gradient)^a^^,b^	254/295	4.7/14.1	30	Stability-indicating method	(8)
UPLC-PDA + Chemometric	BEH C18	MeOH–H_3_PO^4^ (70 + 30)	272	0.32/0.29	<2.5	Co-elution, chemometric resolution	(29)
HPLC	C18	ACN/THF/H_3_PO_4_^c^	275	∼4.6/6.0	>6.0	Conventional method	(30)
RP-HPLC	C18	ACN–1-octane sulphonic acid buffer (0.02 M, pH 3.5) (64 + 36, v/v)	254/295	∼2.5/4.6	>6.0	Validated method	(31)
HPLC	C18	ACN–ammonium formate buffer ? (30 + 70, v/v)	266	0.75/1.5	<3.5	Rapid UPLC method	(9)
UPLC-PDA + DoE (ICRF)	C18	Optimized: ACN– 4 × 10^−⁴^ M CCl₃COOH (60 + 40, v/v)	275	1.62/1.86	<3.0	Fast, resolved peaks, optimized via ICRF	This method

a MeOH = Methanol.

b ACN = Acetonitrile.

c THF = Tetrahydrofuran.

## Conclusions

A UPLC–PDA method for the simultaneous quantification of DH and TI in an ophthalmic formulation was successfully developed using a BBD-based chemometric optimization strategy. Implementation of the ICRF as a composite response variable enabled systematic control of both separation quality and analysis time, resulting in high-resolution chromatographic performance within a short runtime. The optimized method achieved complete separation within 3 min and exhibited excellent linearity, accuracy, precision, and sensitivity. Validation studies confirmed its reliability for quantitative determination, and successful application to a commercial ophthalmic formulation demonstrated its suitability for routine QC analysis. Overall, the results obtained in this study suggest that ICRF-based multivariate optimization can provide a useful approach for simultaneous control of chromatographic resolution and analysis time. Within the investigated experimental domain, the proposed approach yielded chromatographic conditions comparable to those obtained by desirability-based evaluation while providing additional flexibility through adjustable weighting coefficients incorporated into the ICRF framework.

## Author contributions

Erdal Dinç (Conceptualization [Equal], Data curation [Equal], Investigation [Equal], Methodology [Equal], Supervision [Equal], Writing—original draft [Equal], Writing—review & editing [Equal]), Eda Büker (Investigation [Equal], Supervision [Equal], Writing—original draft [Equal], Writing—review & editing [Equal])

## Supplementary Material

qsag060_Supplementary_Data
