# Supplementary material for: ICRF-assisted Box–Behnken design and optimization for rapid UPLC–PDA determination of dorzolamide hydrochloride and timolol maleate in an ophthalmic preparation
Source: J AOAC Int. 2026 Jun 25;109(6):897–905. doi: 10.1093/jaoacint/qsag060 (PMC13732700; doi:10.1093/jaoacint/qsag060)
Supplement: Supplementary file 1 [file qsag060_supplementary_data.zip › qsag060_Supplementary_Data/aoac-26-0051-File016.docx]

**Novelty Statement**

This study introduces an ICRF-based Box–Behnken optimization strategy for UPLC-PDA method development, explicitly incorporating runtime into the multivariate optimization criterion. To the best of our knowledge, no previous study has reported the application of this composite response-based approach for the simultaneous determination of dorzolamide hydrochloride and timolol maleate in ophthalmic formulations. The proposed method provides high-resolution separation within a short 3-minute runtime, enhancing analytical efficiency for routine pharmaceutical quality control.
