# Supplementary material for: ICRF-assisted Box–Behnken design and optimization for rapid UPLC–PDA determination of dorzolamide hydrochloride and timolol maleate in an ophthalmic preparation
Source: J AOAC Int. 2026 Jun 25;109(6):897–905. doi: 10.1093/jaoacint/qsag060 (PMC13732700; doi:10.1093/jaoacint/qsag060)
Supplement: Supplementary file 1 [file qsag060_supplementary_data.zip › qsag060_Supplementary_Data/aoac-26-0051-File017.docx]

June 03, 2026

Manuscript ID: AOAC-26-0051.R2

Title:

"ICRF-Assisted Box–Behnken Design and Optimization for Rapid UPLC-PDA Determination of Dorzolamide Hydrochloride and Timolol Maleate in an Ophthalmic Preparation"

Dear Professor Mengliang Zhang,

Thank you and the reviewers for the valuable comments and suggestions.

We have carefully revised the manuscript and addressed all comments raised during the review process. In particular, a supplementary desirability-function-based evaluation has been included, robustness-related statements have been revised to align with ICH Q2(R2) expectations, and limitations regarding the absence of forced degradation studies have been clearly stated. In addition, several statements throughout the manuscript have been moderated to ensure consistency with the available experimental evidence.

All changes are highlighted in the tracked-changes version, and detailed point-by-point responses are provided in the accompanying response document.

We appreciate your consideration and believe that the revised manuscript is now suitable for publication in the Journal of AOAC INTERNATIONAL.

Sincerely,

Professor Erdal Dinç
